# Population genomic analyses reveal that salinity and geographic isolation drive diversification in a free-living protist

**DOI:** 10.1038/s41598-024-55362-5

**Published:** 2024-02-29

**Authors:** Karin Rengefors, Nataliia Annenkova, Joel Wallenius, Marie Svensson, Anke Kremp, Dag Ahrén

**Affiliations:** 1 Department of Biology, Lund University, 223 62 Lund, Sweden; 2 Institute of Cytology of the Russian Academy of Science, Tikhoretsky Avenue 4, St. Petersburg, 194064 Russia; 3 Department of Clinical Sciences, Faculty of Medicine, Lund University, 223 62 Lund, Sweden; 4 Biology Department, Leibniz Institute for Baltic Sea Research Warnemuende, Seestr. 15, 18119 Rostock, Germany; 5 National Bioinformatics Infrastructure Sweden (NBIS), SciLifeLab, Department of Biology, Lund University, Lund, Sweden

**Keywords:** Biogeography, Evolutionary ecology, Freshwater ecology, Molecular ecology

## Abstract

Protists make up the vast diversity of eukaryotic life and play a critical role in biogeochemical cycling and in food webs. Because of their small size, cryptic life cycles, and large population sizes, our understanding of speciation in these organisms is very limited. We performed population genomic analyses on 153 strains isolated from eight populations of the recently radiated dinoflagellate genus *Apocalathium*, to explore the drivers and mechanisms of speciation processes. Species of this genus inhabit both freshwater and saline habitats, lakes and seas, and are found in cold temperate environments across the world. RAD sequencing analyses revealed that the populations were overall highly differentiated, but morphological similarity was not congruent with genetic similarity. While geographic isolation was to some extent coupled to genetic distance, this pattern was not consistent. Instead, we found evidence that the environment, specifically salinity, is a major factor in driving ecological speciation in *Apocalathium*. While saline populations were unique in loci coupled to genes involved in osmoregulation, freshwater populations appear to lack these. Our study highlights that adaptation to freshwater through loss of osmoregulatory genes may be an important speciation mechanism in free-living aquatic protists.

## Introduction

Our understanding of evolution and mechanisms of speciation is largely based on studies of macroscopic and multicellular organisms. However, the vast diversity of eukaryotes is found within the unicellular microeukaryotes, i.e. protists^1^. Nevertheless, there is limited knowledge regarding divergence and ultimately speciation in protists. In contrast to most multicellular eukaryotes, protists usually have extremely large population sizes, short generations, and reproduction is dominated by asexual reproduction. Consequently, effects of genetic drift, bottlenecks, adaptation, as well as migration rates are expected to differ among these major life forms. Importantly, studying speciation in protists will provide clues to eukaryotic evolution and the evolution of multicellularity^2^.

Settling on a species concept and identifying it is challenging in protists. The most common and practical species concept is the morphospecies, which is based on microscopic morphological differences. The small size and limited morphological variation, makes it difficult to distinguish closely related taxa. The biological species concept^3^ is problematic in protists because some species are strictly asexual, and because sexual reproduction, and thus reproductive isolation, are generally very difficult to detect. Sexual events are often challenging to induce in the laboratory and to identify and quantify in the wild. Thus, determining if populations can interbreed and produce fertile offspring^4^, is usually impossible. However, there is some evidence, at least among green algae, diatoms, and dinoflagellates, that a certain genetic difference is correlated with reproductive isolation^5–8^.

The ecological species concept^9^ is useful for microorganisms including protists. This concept defines a species as a lineage which occupies an adaptive zone different from any other lineage in its range, and which evolves separately from lineages outside its range, ultimately leading to speciation. Shapiro et al^10^ concur that speciation (in microorganisms) is largely driven by natural selection, followed by genome divergence due to reduced gene flow in recombining species—or mutations in clonal lineages. Neutral speciation could potentially occur in cases of drift in conjunction with geographic isolation, but has been regarded unlikely for microbes, because their large population sizes presumably preclude genetic drift. In addition, geographic isolation is considered unlikely due to their putative high dispersal capacity. However, physical barriers have been shown to be important in the speciation of the marine phytoplankton *Gephyrocapsa*^11^. Rengefors et al.^12^ argue that bottlenecks may actually occur during protist population minima and when species invade a new habitat. Moreover, population genetic studies in protists indicate that gene flow among populations is often quite low (see review^12^), which may be enough to promote speciation^13^.

**Figure 1:**
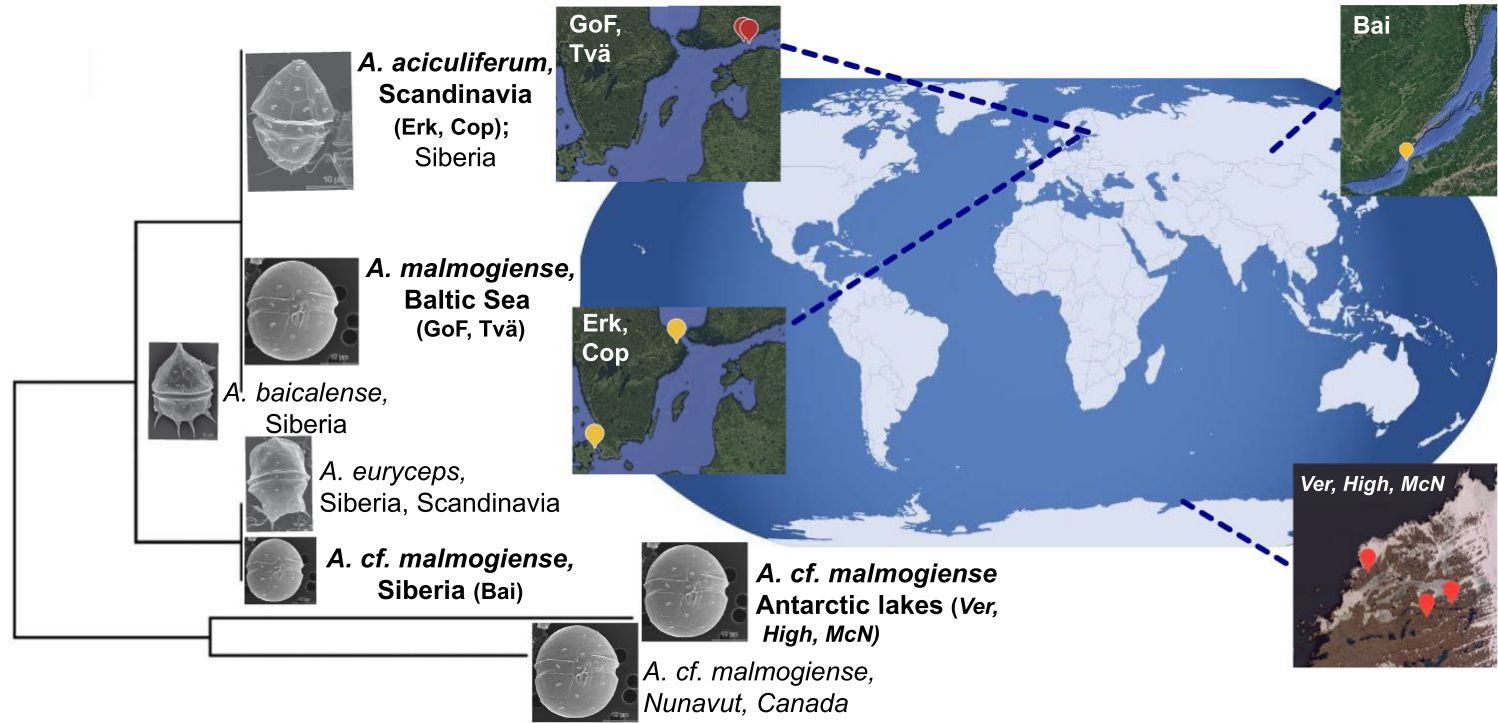
Phylogenetic and geographic distribution of the dinoflagellate *Apocalathium*. The phylogenetic tree of the dinoflagellate genus *Apocalathium is* based on ITS2-LSU rRNA (from Annenkova et al. 2015). Sampling locations of *A. malmogiense* were in the Baltic Sea, *A.* cf *malmogiense* in Siberia (Lake Baikal), *A. aciculiferum* in freshwater lakes in Scandinavia, and *A*. cf *malmogiense* in Antarctic lakes of the Vestfold Hills. Localities: Hig = Highway Lake, ver = Lake Vereteno, mcn = Lake McNeil, gof = Gulf of Finland, tvä = Tvärminne, cop = Sankt Jorgens Sjø in Copenhagen, erk = Lake Erken, bai = Lake Baikal. The figure was made using the world map image accessible under the Creative Commons license (https://wordassociations.net/en/pictures?id=neocreo-Blue_World_Map), and the Google Earth website for the insets (https://earth.google.com/ accessed on 14 September 2023). «Inkscape» (https://inkscape.org/) free graphics editor was used to edit the images.

In this study, we examined the underlying mechanisms that have led to recent speciation in a protist species flock, utilizing a population genomic approach combined with transcriptome data. This species flock consists of closely related lineages of the genus *Apocalathium*, a planktonic, phototrophic dinoflagellate^14^. They occupy similar ecological niches (cold-water, mostly under ice), but which differ in salinity ranging from freshwater to fully marine systems, imposing huge differences in osmotic stress. The genus occurs in geographically widely separated habitats including the two polar zones, lakes as well as the ocean^15^ (Fig. 1). *Apocalathium* consists of four different morphospecies; a rounded type (*A. malmogiense*) and a flattened small-spined type (*A. aciculiferum*), a large spined morphotype (*A. baicalense*) and a large flattened morphotype (*A. euryceps)*. Significant changes in their morphology during the culturing or intermediate forms have not been found^15^. Interestingly, *A. malmogiense*, *A. aciculiferum A. baicalense*, and *A. euryceps* are found sympatrically in ancient Lake Baikal. The different morphospecies have identical 18S rRNA gene sequences but with small differences in LSU and ITS rRNA sequences. Phylogenetic analyses cannot delimit these four morphospecies and gene trees are inconsistent, likely reflecting a recent and rapid adaptive radiation in *Apocalathium*^15^. The secondary structure of the ITS-2 rRNA region, shows that Antarctic and all the other lineages form two separate clusters, suggesting that the Antarctic lineage is reproductively isolated^8^. In contrast, a phylotranscriptomic 792-gene analysis using three strains, showed that the Baltic Sea strains were more closely related to the Antarctic strains rather than the neighboring Swedish freshwater strains^16^. The latter indicates that environment (salinity) could be an important driver in the speciation of *Apocalathium*.

The specific aims of this work were thus to (1) determine genetic differentiation among populations of *Apocalathium* in relation to morphospecies and origin, (2) determine whether geographic isolation or salinity was the most important segregating mechanism, and (3) explore differences in expressed genes between freshwater and saline lineages. Our approach was to perform a population genomic study by generating high-throughput sequencing data in a large number of strains from multiple sites where *Apocalathium* occurs. The resulting data were used to determine population genetic structure, gene flow, and to identify loci that differ between freshwater and saline populations.

## Results

We sampled *Apocalathium* at eight different locations representing two different morphospecies, different habitats (freshwater and saline), and geographic locations (Scandinavia, Baltic Sea, Siberia, and Antarctica) (see “Methods”, Fig. 1, Supplementary Table 1). Two Scandinavian freshwater lake populations belonged to the morphospecies *A. aciculiferum*. All other populations belonged to the morphospecies *A. malmogiense*. Multiple single-cell isolates were cultivated from each location for population genetic analyses. Due to a large genome (~ 30 Gbp) and no reference genome, standard Restriction-site Associated DNA (RAD) sequencing^17^ was applied to obtain Single Nucleotide Polymorphism (SNP) markers. In total, 153 strains, from 8 different locations, were sequenced, and a final 345 shared SNP loci were used for population genetic analyses (see “Supplementary information”).

### Variable within-population genetic diversity

To gain insight into the evolutionary histories of the different populations we calculated various within-population metrics. The highest number of total and variant shared RAD loci were found in the Antarctic and Baltic populations, while the Lake Baikal population had the highest number of private loci, suggesting a more independent evolutionary track (Table 1). Lake Baikal is in fact by far the oldest lake with its 25 million years, and the dinoflagellate populations there are hypothesized to have colonized 5 million to 12,000 years ago^18^. In contrast, the other two freshwater lakes must have been colonized after the last glaciation, so less than 20,000 years ago^19^, while Lake Baikal was not frozen during the last ice age^20^. The lowest genetic variation (nucleotide diversity and population heterozygosity) was found in the Antarctic populations, possibly reflecting a more recent colonization, followed by genetic drift and isolation. The Antarctic lakes are estimated to have been isolated from the sea only ~ 6000 years ago^21^ and are ice-covered most of the time, likely minimizing new immigrations^22^. Nucleotide diversity was highest in the Lake Erken population, as was population heterozygosity (Table 1), despite that the lake is estimated to have formed 3000 years ago, separating from the Baltic Sea^23^ following land-rise.

**Table 1:** Summary statistics from Stacks-population runs of all populations.

Population	Total RAD sites	Variant sites	Private loci	Nucleotide diversity (π)	Nei total heterozygosity
SCA-Copenhagen	29 255	237	70	0.0154	0.055
SCA-Erken	11 914	96	25	0.1691	0.176
SIB-Baikal	27 601	222	92	0.0532	0.056
BAL-Gulf of Finland	38 224	313	6	0.0123	0.001
BAL-Tvärminne	38 342	314	6	0.0211	0.013
ANT-Highway	41 517	338	2	0.0007	0.001
ANT-McNeil	39 994	328	2	0.0005	0.022
ANT-Vereteno	41 527	340	2	0.0021	0.002

Data reported includes total number of shared RAD sites (variant and fixed), the number of variant loci, the number of private loci, nucleotide diversity (π), and population-level heterozygosity from Genodive. Populations were labeled by the regions and sampling locations. The regions include Scandinavia (SCA), Siberia (SIB), Baltic Sea (BAL), Antarctica (ANT).

### High genetic differentiation among populations

**Figure 2:**
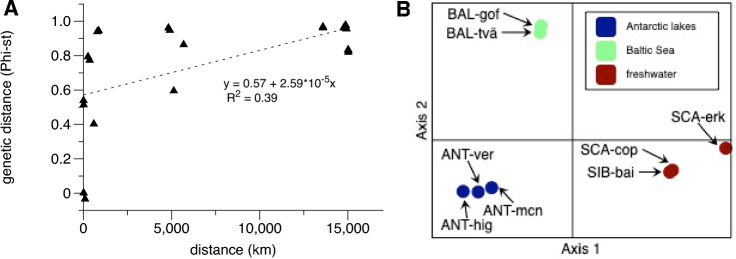
*Apocalathium* population genetic isolation-by-distance and clustering. (**A**) Pairwise comparisons of lake populations with geographic distance (km) plotted against genetic distance (Phi-st). Dotted line is regression and R^2^ correlation value. (**B**) Principal Correspondence Analysis based on SNP data of eight populations of *Apocalathium* from saline Antarctic lakes (dark blue), the Baltic Sea (light green) and freshwater lakes (dark red). Regions: ANT = Antarctica, BAL = Baltic Sea, SCA = Scandinavia, SIB = Siberia. Localities: hig = Highway Lake, ver = Lake Vereteno, mcn = Lake McNeil, gof = Gulf of Finland, tvä = Tvärminne, cop = Sankt Jorgens Sjø in Copenhagen, erk = Lake Erken, bai = Lake Baikal.

Pairwise comparisons of dinoflagellate populations from all eight locations showed high and significant differences between all geographic regions (Phi_ST_ values between 0.82 and 0.97; Table 2). In contrast, within the Baltic Sea, the two populations Tvärminne and Gulf of Finland, which are hydrologically connected, were not significantly differentiated. Similarly, the Antarctic populations in lakes Highway and McNeil, which are less than 10 km apart, were not significantly different. However, genetic distance was not always correlated with geographic distance, and while the Mantel test of Isolation-By-Distance (IBD) showed significant (*p* = 0.001) genetic isolation with geographic distance, geographic distance only explained 38.9% of the variation (Fig. 2A). For instance, the Antarctic Lake Vereteno population was significantly different from the other two Antarctic populations (Phi_ST_ = 0.5) despite close geographic proximity (< 10 km). The two lake populations of the freshwater *A. aciculiferum* had a relatively high Phi_ST_ of 0.4 although the geographic distance was moderate (587 km).

**Table 2:** Population differentiation versus distance.

	SCA-Copen-hagen	SCA-Erken	SIB-Baikal	BAL-Gulf of Finland	BAL-Tvärminne	ANT-Highway	ANT-McNeil	ANT-Vereteno
SCA-Copenhagen		0.403	0.864	0.945	0.940	0.963	0.962	0.962
SCA-Erken	587		0.595	0.774	0.794	0.835	0.820	0.830
SIB-Baikal	5682	5122		0.961	0.948	0.968	0.962	0.963
BAL-Gulf of Finland	864	349	4819		− 0.034^n.s^	0.978	0.976	0.974
BAL-Tvärminne	785	259	4898	91		0.959	0.955	0.955
ANT-Highway	14,769	15,019	13,586	14,868	14,906		0.002^n.s^	0.514
ANT-McNeil	14,779	15,028	13,591	14,878	14,915	9		0.539
ANT-Vereteno	14,779	15,028	13,588	14,878	14,915	10	2.5	

Pairwise genetic population differentiation (Phi_ST_) of populations from all eight sites above the diagonal. Pairwise geographic distance (km) below the diagonal. All differences except those with n.s. as superscript are significant.

Although there is no pre-determined cut-off for Phi_ST_ values representing different species, a value of 1 means no gene flow. The Antarctic populations of *A.* cf *malmogiense* had extremely high Phi_ST_ values close to 1 (0.82–0.978) in all pairwise comparisons to other geographic locations, regardless of morphospecies or habitat. This provides strong support that the Antarctic lineage should be considered a distinct species. Similarly, the Baltic Sea lineage, has high pairwise Phi_ST_ with all other lineages, including the geographically close *A. aciculiferum* populations.

The Phi_ST_ data does not support that populations of the same morphospecies are more similar to each other than different morphospecies. For example, *A.* cf *malmogiense* from Lake Baikal was more similar to the other the Swedish Lake Erken *A. aciculiferum* (Phi_ST_ = 0.595) than to the other *A. malmogiense* populations.

### Population structure analyses suggests three to four major lineages

**Figure 3:**
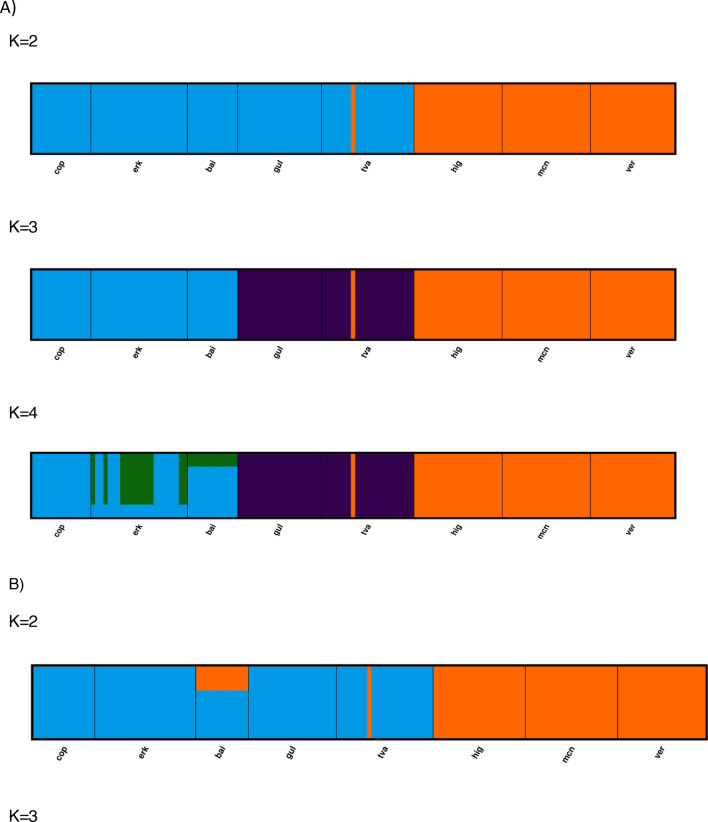

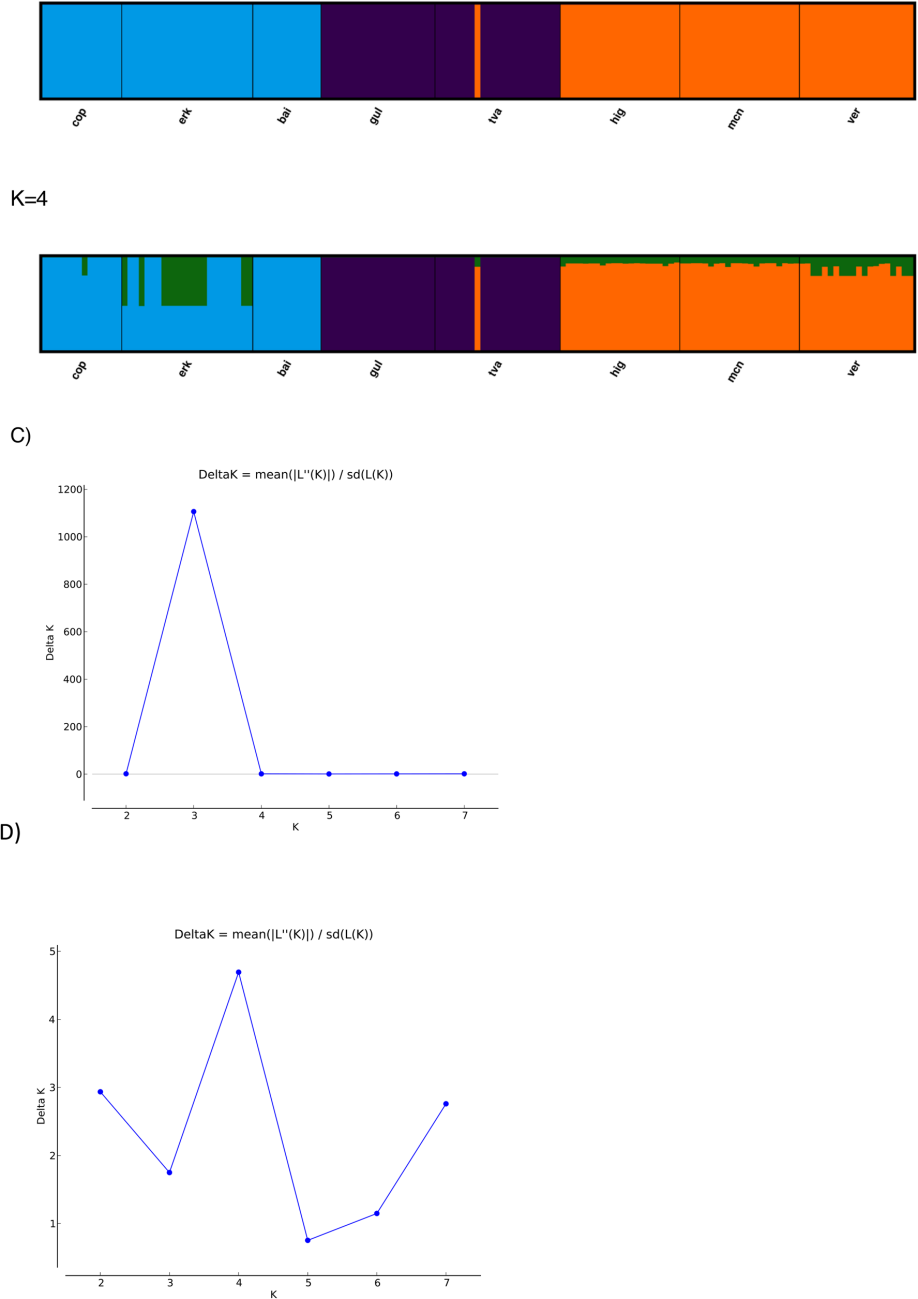
*Apocalathium* population Structure analysis based on 345 SNPs. Each color represents a putative population. K denotes the number of putative populations. (**A**) Model allowing no admixture and independent alleles for putative number of populations varied between K = 2–4. (**B**) Model allowing admixture and independent alleles showing putative number of populations varied between K = 2–4. (**C**) Evanno plot showing that the delta K value for (**A**) is highest for K = 3. (**D**) Evanno plots showing that the deltaK value for (**B**) is highest for K = 4. hig = Highway Lake, ver = Lake Vereteno, mcn = Lake McNeil, gof = Gulf of Finland, tvä = Tvärminne, cop = Sankt Jorgens Sjø in Copenhagen, erk = Lake Erken, bai = Lake Baikal.

Together the different clustering and population structure analyses all point towards the separation of three to four genetic lineages. The PCoA showed that the populations clustered in 3–4 groups; Antarctic lakes, Baltic sites, and the freshwater lakes form three major clusters (Fig. 2B). Axis 1 and 2 explained up to 87.41% of the variation. Within the freshwater group, populations were more separated than in the other two groups (i.e. Baltic Sea, Antarctic lakes), suggesting that the Lake Baikal population could be a fourth genetic group. The AMOVA indicated that most of the genetic variation was within populations (54%), while 32% was among the groups identified in the PCoA, and only 14% among populations. The STRUCTURE analysis also identified three distinct populations to best describe the data when the no-admixture model was used (i.e. no current gene flow assumed), regardless of the allele model (Fig. 3). These consisted of the Antarctic populations in one, the Baltic Sea populations in a second, and the freshwater populations in a third population. When allowing for admixture, four populations best explained the data, but the fourth population was admixed in all sites and did not overlap with a separate Lake Baikal population, thus not adding information on population structure (Fig. 3). When a K-clustering analysis was performed (see “Methods”), 6 populations were detected, where each geographic site was one separate population, except for all three Antarctic lakes which formed a single population.

### Unique loci when contrasting freshwater and saline populations

To explore differences between freshwater populations and those adapted to brackish saline water habitats, we identified unique RAD-loci to each of those groups (“Supplementary information”, Methods). The RAD sequences representing these loci were then mapped against a merged transcriptome database consisting of 506,560 reads from strains originating from freshwater Lake Erken (cultivated at salinities 0 and 3) and saline Baltic Sea and Highway lake (cultivated at salinities 0, 3 and 30). From the unique loci that had a transcriptome hit and a hit against SwissProt, the top loci differentiating freshwater vs saltwater populations were analyzed further in terms of gene ontology (GO). When comparing presence in salt- but not freshwater this yielded 90 different GO terms (Supplementary Table 2), while for presence in freshwater, but not saltwater, accounted for only 6 GO terms (Supplementary Table 2). Saltwater GO-loci were connected to chloride channels, iron transmembrane transport activity, and divalent cation transport, all involved in osmoregulation. Several loci were connected to betaine glycine and glyceraldehyde-3-phosphate dehydrogenase activity, i.e., connected to osmolytes (Table 3). Moreover, loci involved in urea transport as well as cell wall callose deposition were highly represented. GO-loci present in freshwater only were mainly connected to sodium homeostasis and citrate transport (Table 3).

**Table 3:** Unique RAD loci in freshwater versus saltwater strains. Top table—top 20 gene ontology terms of sequences with matches between RADSeq loci and RNA transcripts that are unique for saline populations. For stringency only GO terms with at least 10 loci were considered. Lower table—Top gene ontology terms of sequences with matches between RADSeq loci and RNA transcripts that are unique for freshwater populations. For stringency only GO terms with at least 10 loci were considered.

GO term	GO annotation	Freshwater counts	Saltwater counts
F:0005247	Voltage-gated chloride channel activity	0	34
F:0005254	Chloride channel activity	0	29
C:0034707	Chloride channel complex	0	25
F:0005381	Iron ion transmembrane transporter activity	0	22
F:0015093	Ferrous iron transmembrane transporter activity	0	22
P:0034755	Iron ion transmembrane transport	0	22
P:0072511	Divalent inorganic cation transport	0	22
C:0033573	High-affinity iron permease complex	0	20
P:0006268	DNA unwinding involved in DNA replication	0	20
F:0008886	Glyceraldehyde-3-phosphate dehydrogenase (NADP+) (non-phosphorylating) activity	0	19
P:0031460	Glycine betaine transport	0	17
P:0009115	Xanthine catabolic process	0	17
C:0033177	Proton-transporting two-sector ATPase complex, proton-transporting domain	0	17
F:0004855	Xanthine oxidase activity	0	17
C:0033179	Proton-transporting V-type ATPase, V0 domain	0	17
P:0071705	Nitrogen compound transport	0	17
P:0009863	Salicylic acid mediated signaling pathway	0	17
F:0004854	Xanthine dehydrogenase activity	0	17
F:0043047	Single-stranded telomeric DNA binding	0	16
P:0015847	Putrescine transport	0	16

GO term	GO annotation	Freshwater counts	Saltwater counts
P:0075522	IRES-dependent viral translational initiation	24	0
P:0032790	Ribosome disassembly	24	0
P:0003091	Renal water homeostasis	14	0
P:0003096	Renal sodium ion transport	12	0
P:0055074	Calcium ion homeostasis	12	0
P:0035812	Renal sodium excretion	12	0

## Discussion

Using population genomics, we found strong support for the hypothesis that the environment, specifically salinity, is a major factor in driving ecological speciation in the dinoflagellate *Apocalathium*. Geographic isolation also plays an important role, showing unambiguously that protists do not disperse at a higher rate than the rate of genetic differentiation, thereby allowing for allopatric speciation. In addition, we found that morphological similarity is not equivalent to genetic identity, demonstrating that the morphospecies concept is not suitable for all protists.

A key finding in our study was that the genomic data did not support the current division of *Apocalathium* into two morphospecies, but rather into four lineages or species: (1) *A. malmogiense* from the Baltic Sea (2) *A*. cf *malmogiense* from the Antarctic lakes, (3) freshwater *A. aciculiferum*, and (4) *A.* cf *malmogiense* from Lake Baikal. Clearly the overall cell morphology is not a good species delineator in *Apocalathium*. The high pairwise Phi_ST_ values, which are close to 1, also support that these are four different species. Moreover, the fact that only 345 loci (out of 264,000 per individual) were shared under relatively relaxed parameters, further reflects on the large differences among these populations. Interestingly, *A. aciculiferum* from Scandinavia and *A.* cf. *malmogiense* from Lake Baikal clustered together despite having different morphologies. Possibly the round morphology confers some advantage in the sea and “sea-like” habitats like Lake Baikal, which with its huge depth and volume is more like a sea than a lake. Alternatively, cell morphology is a non-adaptive trait and the two lineages have different evolutionary trajectories.

A striking result was the distinct genetic structure and overall high level of genetic isolation among populations. Within the distinct lineages, there was either no significant differentiation (e.g. Baltic *A. malmogiense* populations which are hydrologically connected), or moderate differentiation (*A. aciculiferum* populations, Antarctic *A.* cf*. malmogiense*). Surprisingly, some pairwise lake population comparisons within the same lineage had a relatively high Phi_ST_ (0.4–0.5) suggesting limited gene flow and rapid differentiation in lake populations. Previous studies using Amplified Fragment Length Polymorphism (AFLP) and microsatellites (reviewed in^12^) have also shown that phytoplankton lake populations typically are significantly genetically differentiated. Even populations of phytoplankton in the Baltic Sea show fine-scale differentiation despite currents connecting the water masses^24–26^. While the mechanisms for this differentiation within species is not well understood, there is clearly limited gene flow among phytoplankton populations despite potentially high dispersal capacity. It has been suggested that monopolization of first colonizers^27,28^ together with anchoring through resting cyst seed banks^29^ contribute to limited gene flow.

Our results suggest that speciation has not been driven by geographic isolation primarily in *Apocalathium*. While genetic differentiation among populations was always high at all the large distances (> 1000 km), they varied considerably at smaller distances. Also, although the clustering analysis mostly separated geographically distant populations, the freshwater Baikal strains clustered with Scandinavian freshwater populations despite being geographically very distant. We interpret this as follows: while geography plays a certain role in protist speciation, local processes, both within lakes and among habitats are more important. This finding is in line with other population genetic studies of limnic populations, demonstrating that isolation-by-distance falls apart at a certain distance^27^.

Here we provide evidence for ecological speciation, driven by habitat salinity, that has led to differentiation of freshwater and marine-brackishwater lineages. Salinity has previously been hypothesized as a major driver in the speciation of protists in general^30^ including *Apocalathium*^31^. Recent work has demonstrated that freshwater-marine transitions are not as infrequent as proposed earlier, but that freshwater and saline species form phylogenetically distinct groups^32^. Our comparison of the freshwater versus the saline lineages indicate that the lineages have different sets of genes. Markedly, multiple unique loci in populations from saline habitats were found for genes related to osmoregulation. Several of these were involved in transport and catabolic processes of urea and glycine betaine, which are natural osmolytes that can serve as osmoprotectants^33^. By accumulating osmoprotectants the cell can balance the osmotic stress between the cell and the surroundings, and thereby maintain cell turgor and volume^33^. Glycine betaine has been found in various marine dinoflagellates associated with increased salinity^34–36^. Moreover, in a transcriptome analysis of the dinoflagellate *Oxyrrhis marina*, genes related to the glycine betaine pathway were upregulated when cells were grown in extremely high salinity (50 psu)^37^. In addition to genes related to glycine betaine, there were multiple hits related to chloride channel and sodium transporters. In mammalian cells, maintenance of cell volume is regulated by Na^+^/Cl^−^ transport across the cell membrane, where shrinkage is counter-acted by accumulation of ions by Na^+^, K^+^, and 2Cl^−^ transport. Thus, these transcripts are probably also utilized by *Apocalathium* to maintain cell volume.

While populations from saline water had unique RAD-loci with hits against osmoprotectant-related processes, no such hits were found for the freshwater populations. Instead, the most frequent unique loci were found in genes coupled to regulation of calcium and sodium homeostasis. In freshwater environments, cells experience a hypotonic exterior environment, and water rushes into the cell causing swelling. Eukaryotic cells have either evolved aquaporins or contractile vacuoles to channel out water. However, contractile vacuoles are absent in dinoflagellates. Instead they have a pusule system which likely takes part in osmoregulation via a different mechanism^38^. Klut et al.^39^ showed that the dinoflagellate pusule structure is a fibrillar collar system which together with the flagella may be connected to water expulsion. Surprisingly, further studies regarding osmoregulation of freshwater dinoflagellates are lacking. While speculative, the unique hits for freshwater lineages were connected to calcium and sodium homeostasis, as well as renal function-associated genes, suggesting that these could be linked to expulsion of freshwater.

Interestingly, transcripts connected to osmoregulation (glycine betaine, Na^+^/Cl^−^ transport) were found in saline lineage transcriptomes (*A. malmogiense,* Antarctic *A cf. malmogiense*), but were lacking in the freshwater (*A. aciculiferum*) transcriptomes. This means that these genes are either not transcribed, found in very low copy number, or are not present at all, in the freshwater strains. To verify if the genes are still present in the genome of freshwater lineages in-depth genome sequencing is needed. However, since most dinoflagellates have constitutively expressed genes, a lack of transcription is less likely an explanation for the majority of the genes^40^. This is corroborated by the fact that in the laboratory *A. aciculiferum* grew at 0 and 3 psu but was unable to sustain growth at 30 psu^31^. In contrast, the genes related to putative freshwater expulsion, were found in the transcriptomes of all three lineages, even if the RAD-loci hits were unique for freshwater strains. Since saline lineages grew at 0 psu^16^, these lineages must have retained the ability to pump out water to maintain cell turgor. Since the RAD-loci were unique to freshwater strains, this suggests that there are genetic differences between the two groups in these genes. A plausible explanation is that there is variation in gene copy number, and that saline lineages have much fewer copies than freshwater lineages, resulting in few RAD-loci which are lost in the bioinformatic filtering. Dinoflagellates are known to have high gene copy numbers, often structured in tandem repeats, with within copy variation^41^. Another possible, but perhaps more unlikely explanation is that there are SNPs in the SbfI cut-site in the saline strains, thereby removing these RAD sites.

The lineages belonging to *Apocalathium* have previously been proposed to have undergone a recent adaptive radiation^15^. The current study supports this hypothesis since the differences between the freshwater and saline populations are not only found in neutral SNPs but also in functional genes. Given that there are multiple genes related to osmoregulatory capacity, this supports the hypothesis that the divergence is adaptive. We hypothesize that the ancestral *Apocalathium* species was a cold-water euryhaline marine species and that the freshwater species evolved when trapped in glacial lakes following recession of glaciers. However, the *A.* cf *malmogiense* in Lake Baikal may have evolved earlier since Lake Baikal was not frozen during the last glaciation^20^. Following adaptation to freshwater, the limnic lineages appear to have lost their ability to osmoregulate in water with salinity more than 3. This scenario may also explain the speciation of the closely related dinoflagellates *Gymnodinium baicalense* and *G. corollarium*, which inhabit the Baltic Sea and Lake Baikal respectively, and which differ in their ability to grow in saline water^42^. Thus, loss of osmoregulatory genes or switching off of their expression may be an important mechanism in speciation of protist that have transitioned between marine and freshwater environments.

Our study revealed that RAD-sequencing is both a feasible and successful strategy for population genetic/genomic studies in dinoflagellates, and that in combination with transcriptomes can provide functional information on loci of interest. Given the size of the *Apocalathium* genomes the initial concern was that RAD-seq would be unfeasible. Using an 8-cutter restriction enzyme such as SbfI a total of 916,000 RAD sites were estimated, but around 264,000 were recovered on average per individual. A plausible explanation is that dinoflagellate genomes contain a large fraction of repetitive elements, being as high as 68% in the polar *Polarella glacialis*^40^. Despite this high number of RAD-sites we were able to sequence enough to have a high coverage per RAD-site. However, the loss of RAD-loci was high when filtering for shared loci (see “Supplementary Information”). We interpret this loss to be due to the large genome size (unequal sequence depth) and high diversity. Nevertheless, sufficient SNPs were recovered to perform the study, making RAD-seq an excellent alternative to whole-genome sequencing which for these organisms is not feasible.

To conclude, in this study we show that salinity is likely an important driver for population differentiation in the dinoflagellate *Apocalathium,* but also that geographic isolation plays an important role. The high genetic differentiation and the presumed loss of multiple genes involved in osmoregulation suggests that these lineages should be considered as separate species that no longer exchange genes. The implications of these results provide evidence of ecological speciation as an important process in the microbial world.

## Methods

### Sampling and isolation of dinoflagellate strains

Strains from the dinoflagellate genus *Apocalathium* were sampled from 8 different locations (lakes and sea) in four different geographic regions (Scandinavia, Baltic Sea, Siberia, Antarctica) (Supplementary Table 1). *Apocalathium* consists of four different morphospecies, where the rounded type (*A. malmogiense*) was described from a pond filled with Baltic Sea water^43^ and is currently found in saline habitats (Baltic Sea, the Arctic Ocean, brackish Antarctic lakes) and in the ancient freshwater Siberian Lake Baikal^15,44^, and a flattened small-spined type (*A. aciculiferum*) is found in northern temperate lakes including bays of Lake Baikal. In addition, a third large spined morphotype (*A. baicalense*) is allegedly endemic to Lake Baikal, and a fourth large flattened morphotype (*A. euryceps*) has been encountered in Swedish freshwater lakes and Lake Baikal, but these are not included in the current study as they could not be cultivated. Strains from the Baltic Sea belonging to the morphospecies *A. malmogiense* were isolated from material collected at Tvärminne Zoological Station (TV), at the south-west coast of Finland in 2009 and 2010, and at the monitoring site LL7 in the Gulf of Finland in 2013. Single motile cells were isolated from net tow samples and cyst from surface sediment slurry incubations into separate wells of a 24-well tissue culture plate containing 1.5 mL enriched sea water (f/8-Si, salinity of 6.5^45^), and incubated at 4 °C, 14:10 light:dark cycle and 100 μmol photons m^−2^ s^−1^.

The Antarctic strains (morphospecies *A.* cf *malmogiense)* were isolated from brackish-saline lakes during an Antarctic expedition in 2009 as described in^22^. Freshwater strains of *A.* cf *malmogiense* from Lake Baikal in Siberia, Russia and *A. aciculiferum* originated from Lake Erken, Sweden, and a pond in central Copenhagen, Denmark, sampled in winter/early spring 2014 (Supplementary Table 1). Three strains from lake Erken were isolated in 2004. Cells from the freshwater lakes and Antarctic lakes were isolated from plankton samples collected with a 20 µm net. Individual cells were isolated manually, washed three times, and transferred to separate wells of a 48-well tissue culture plate. For the freshwater strains the wells contained 50% sterile-filtered lake water and the remainder artificial MWC medium with a selenium (Se) amendment (see^46^). Cultures were grown at 4 °C in at 12:12 LD cycle at 50 µmol photons m^-2^ s^-1^. When cultures had been established, they were further grown in MWC + Se only. The Antarctic strains were first isolated as described in^22^. The strains were subsequently transferred to f/2 medium with salinity 7–8, achieved by diluting sterile-filtered seawater with MQ water.

### DNA extraction and RAD library preparations

All samples were harvested in 2015 by spinning down 30 ml culture in mid-late exponential phase for 10 min at 2000 g. DNA extractions were performed using the Qiagen DNeasy Plant Mini Kit (Qiagen) and DNA was quantified by Qubit. We followed a RAD library preparation protocol modified from^47^ and^17^ described in^48^. For each sample, 1 µg of genomic DNA was digested with 0.5 µl SbfI-HF (NEB, Ipswich, MA, USA). We used 0.5 µl of 2000 U/µl T4 ligase (NEB) in the P1 and P2 adapter (for sequences see “Supplementary Information”) ligation steps and decreased the volume of NEB2 buffer (1 µl) used in the P1 adapter ligation. P1 adapters contained unique 7 bp barcodes to allow multiplexing strains in downstream library preparation, and 3 µl of barcoded P1 adapter (100 µM) were used in each ligation reaction. The final full amplification was performed with 67 ng of DNA template in a 100 µl reaction volume and 18 PCR cycles. The 300–700 bp size fraction of the PCR product was excised and purified from an agarose gel. 20 uniquely barcoded strains were pooled per lane for sequencing in order to recover at least 8 million reads per sample, meaning at least 40 × coverage. Samples were sequenced with Illumina technology at the SNP&SEQ Technology Platform of the SciLifeLab facility in Uppsala, Sweden. Sequencing was performed using Illumina HiSeq2000 v4-chemistry, 125 bp. The R2 reads were not used in the downstream analyses. These sequences have been submitted to BioProject: PRJNA1025931.

### RAD/SNP identification

All data was de-multiplexed, quality-checked, and processed using the Stacks software version 1.35 https://catchenlab.life.illinois.edu/stacks/^49,50^. The analysis pipeline was run manually. Following ustacks which builds loci, the number of retained sequences, RAD tags, and SNPs per sample were collected. Stacks software parameters were tested with a pilot data set using four strains with 4 M reads each. The parameters were chosen with the criteria to maintain a mean coverage of at least 30, and maximize number of utilized reads and polymorphic SNPs, by varying mismatch (M 0–1) and depth of stack (m 3 to 5) parameters. The final Stacks pipeline run was set with ustacks having the parameters -m 5 -M 0 -N 1 to build the RAD-locus catalog. The cstacks step was run with the number of mismatches (n) allowed between sample tags when generating the catalog, set to 2. For further details regarding choice of Stacks parameters see^51,52^.

Prior to proceeding with downstream analyses potential bacterial contaminant sequences were removed. This was done using the taxonomic sequence classifier Kraken2, version 2.0.8-beta^53^ to identify and subsequently blacklist those loci. This was done following steps 2 and 3 on https://github.com/DerrickWood/kraken2/wiki/Manual#custom-databases using the library *“bacteria: RefSeq complete bacterial genomes/proteins”*. All diploid loci were also identified and blacklisted to only retain haploid loci (since most dinoflagellates are haploid). Before filtering, a total of 6,450,531 RAD-tag loci were identified in the data set with a mean of 219,299 per individual. Of these, 3.3% were classified as bacterial by Kraken. Only the first SNP in each RAD-tag was used for further analyses to avoid linked loci and hereafter referred to as RAD-loci.

### Selection of loci

From the total number of RAD-loci in the Stacks catalog, a subset of loci was selected for further analysis. Selection was based on the parameters p and r in Stacks-population, where p represents the number of populations that must contain a locus, and r is the proportion of individuals per population that must have that locus. The total number of common loci were identified for different p and r parameters (“Supplementary Information”, Table 1).

In addition to the standard of 8 different populations inhabiting geographically distinct locations, different a priori population divisions were explored using the popmap feature in Stacks-population. These were divided into the following groupings: *salinity* (2 populations): saline (all Antarctic + Baltic Sea strains) and freshwater (Scandinavian lakes, Lake Baikal); *species* (3 populations): *A. malmogiense* (Baltic + Antarctic), *A. aciculiferum* (Scandinavian lakes), *A.* cf *malmogiense* sp*.* (Lake Baikal); *geography* (4 populations): Antarctic lakes, Scandinavian lakes, Baltic Sea, and Lake Baikal. For these, p and r parameters were also varied (Suppl. Table 1). With a separate python script (https://github.com/Jolleboll/Dinoflagellates/blob/main/scripts/common_loci_among_samples.py) shared and unique loci were selected from the RAD catalog, and subsequently enumerated, listed, and used for each pairwise comparison among populations. This was done for each of the population divisions described above.

### Shared loci in Antarctic *A. malmogiense* populations

Shared loci among populations in close geographic vicinity of each other was explored by focusing on the subset including only the individuals from the Antarctic lakes. Shared loci, i.e. loci that were found in all three populations were utilized. The percentage of individuals containing the locus was set to vary between 20 and 80%.

### Mapping of all catalogued loci to transcriptomes

To explore differences among populations in functional regions of the genome, the RAD-tag loci identified were mapped against transcriptomes of the studied populations. Transcriptomes of one strain each from *A. malmogiense* (Baltic Sea, Tvärminne), *A.* cf. *malmogiense* (Antarctica, Highway Lake) and *A. aciculiferum* (Freshwater, Lake Erken) were available from the MMETSP project^54^. The transcriptomes had been extracted from monoclonal cultures grown at three different salinities at 4 °C: 0, 3, and 30, except for *A. aciculiferum* which was grown at salinities 0 and 3, because it was unable to grow in more saline conditions. In short, 4 L of exponentially growing cultures were harvested by centrifuging 10 min at 1000 g. The supernatant was removed and the pellet was stored at − 80 °C until extraction. Total RNA was extracted using Qiagen RNeasy Plant Mini Kit (Qiagen), resulting in a final amount of 3 µg RNA per sample. All further processing and sequencing were performed by the MMETSP project according to protocols found in^54^. The available 8 transcriptomes were merged by morphospecies, with 3 of each except for *A. aciculiferum* which consisted of 2 transcriptomes.

### Population genetic metrics

SNP-loci found in at least 6 of the 8 populations and in at least 50% of the individuals in a population (set within the Stacks population program) were selected for downstream population genetic analyses. The reason for using these more relaxed parameters is that we were dealing with at least two and possibly multiple species. Moreover, dinoflagellates have very large genomes, which may be the cause for the high loss of loci when p and r are constrained, even within a species. To maximize loci, yet at the same time include most locations, and a reasonable number of individuals, we decided to stick with p = 6 and r = 0.5. All outfiles were formatted directly in Stacks Population, or converted from the Stacks output utilizing PGDspider 2.1.1.5 http://popgen.unibe.ch/software/PGDSpider/. Population differentiation (F_st_), and genetic diversity (π) were calculated directly in Stacks based on all SNPs utilized by the Stacks population program. Data on number of RADsites, variant alleles, and polymorphic sites were also obtained. GenoDive 2.0b27 https://www.patrickmeirmans.com/software/GenoDive.html^55^ was used to calculate Phi_ST_, Nei’s genetic diversity and isolation-by-distance (IBD, Mantel test).

### Population structure analyses

A Principal Coordinates Analysis (PCoA), based on PhiPT was run in GenAlEx6.51b2 https://biology-assets.anu.edu.au/GenAlEx/^56,57^, to determine the major pattern of the data. Partitioning of genetic variance was performed using an AMOVA, also in GenAlEx6.51b2, partitioning the data into the 8 populations, as well as three regions (Antarctic, Baltic Sea, and Freshwater lakes). Population genetic structure was inferred using a Bayesian analyses in the software Structure v. 2.3 https://web.stanford.edu/group/pritchardlab/structure.html^58,59^. 153 individuals from all eight populations and 345 variant loci were utilized. The number of putative populations (K) was set from 1 to 8, with 10,000 burn-ins and 20,000 MCMC repetitions, and 5 iterations. The parameters chosen were admixture and correlated alleles, admixture and independent, no admixture and correlated, and no admixture independent alleles. Estimates of the best K to describe the data was determined using the software Structure Harvester with the Evanno method https://github.com/dentearl/structureHarvester/^60^ as well as manual visual inspection. Population structure was also determined using a k-clustering analysis with the software Genodive, as this method makes no assumptions about Hardy–Weinberg equilibrium. Clustering was performed from 1 to 6 clusters using both allele frequencies and an AMOVA approach, with 50,000 annealing steps and 20 random starts.

### Loci under selection

Loci used for population metrics (i.e. all 8 populations with Stacks-population parameters p = 8 and r = 0.3) were used to check for loci under selection. These parameters were utilized in order to make sure the loci were present in all 8 populations (p = 8) yet have enough loci to analyze (r therefore set to 0.3). The structure output file was converted using PGDSpider and subsequently used for the software BayeScan 2.1 http://cmpg.unibe.ch/software/BayeScan/. Loci under strong selection pressure had their RAD-sequences blasted against a non-redundant database formed by merging the 8 transcriptomes (ID lines were concatenated).

### Unique loci when contrasting freshwater and saline populations

To explore differences between freshwater populations and those adapted to brackish saline water habitats, we identified unique loci with annotated matches. We utilized the population division “salinity” (see above). Loci that were unique to one population (either freshwater or saline) were saved as fasta format and then blasted against the merged transcriptome. For all blasted loci, the top hit's top annotation's GO terms were tallied across loci to a per-population total. Loci whose top hits lacked annotation were ignored, as were loci without blast hits. A subset of the GO-terms were unique in one of the two populations. These were selected for in-depth gene ontology analysis, to determine potential function of involved transcripts. The threshold values for further evaluation were set to at least a tenfold difference between freshwater and saline.

### Supplementary Information

Supplementary Information.

## Data Availability

The dataset generated and analyzed in this study are deposited at BioProject: PRJNA1025931 by following the link: https://dataview.ncbi.nlm.nih.gov/object/PRJNA1025931?reviewer=rsm9mavsm3nu9airtrnui5q09e.
