# Supplementary material for: Population genomic analyses reveal that salinity and geographic isolation drive diversification in a free-living protist
Source: Sci Rep. 2024 Feb 29;14:4986. doi: 10.1038/s41598-024-55362-5 (PMC10904836; doi:10.1038/s41598-024-55362-5)
Supplement: Supplementary file 1 — Supplementary Information. [file 41598_2024_55362_MOESM1_ESM.docx]

Supplemental Information Rengefors et al.

**Supplemental methods**

P1 Adapter

TOP 5'-AATGATACGGCGACCACCGAGATCTACACTCTTTCCCTACACGACGCTCTTCCGATCTXXXXXXXTGC*A-3'

BOTTOM 5'-/5Phos/XXXXXXXAGATCGGAAGAGCGTCGTGTAGGGAAAGAGTGTAGATCTCGGTGGTCGCCGTATCATT-3'

P2 Adapter

TOP 5´- P-CTCAGGCATCACTCGATTCCTCCGAGAACAA -3´

BOTTOM 5´- CAAGCAGAAGACGGCATACGACGGAGGAATCGAGTGATGCCTGAG*T -3´

**Supplemental Results**

**SNP-metrics.**

SNP-loci were first analyzed within a subset of the populations, which certainly belonged to one species/lineage. For this purpose, we chose the three populations from lakes situated on the Antarctic peninsula. These lakes are less than 10 km apart and all belong to the Antarctic lineage *Apocalathium* cf*. malmogiense*  ^12,13^. The Stack parameter r, i.e. the minimum percentage of individuals with a locus in order to keep a locus was varied between 0.2 and 0.8. A total of 160,953 loci were recovered with r= 0.2 but only 4,262 remained when r= 0.8 (Suppl. Table 1).

Remaining loci shared across species division, geographic areas, or the salinity barrier, further reduced the total number of retained loci (Suppl. Table 3). Across the salinity barrier (data split into two populations) only 123 loci were retained when the minimum percentage of individuals was set to 50%. When species division (*A. aciculiferum, A. malmogiense* and Antarctic *A.* cf *malmogiense*) was used, only 81 loci were retained with r=0.5. Similarly, with geographic division (Antarctica, Scandinavian lakes, Baltic Sea, Lake Baikal) only 61 loci were retained.

For the analyses of all eight sites, with p=8 and r=0.5, only 35 loci were retained (Suppl. Table 3). When p was reduced to 6 and r set to 0.5, 345 variant loci resulted. These 345 loci were used for downstream population genetic analyses.

**Loci under selection**

For the shared vs unique loci analysis, all loci were retained, i.e. no filtration restraints (stacks p and r parameters) were used. Using a population division based on salinity, the number of unique loci for the freshwater group was 2,390,814 and for the saline group it was a total of 4,021,349 loci. In contrast, the number of common loci in freshwater and saline group was only 38,368. The analysis for loci under selection utilizing Bayescan yielded only three loci with a probability score above 90% (out of 734) as being potential loci under selection. Of these three, one mapped against the transcriptome, but unfortunately against a non-annotated region.

Mapping all the RADtag sequences against the Antarctic lineage (*A.* cf *malmogiense*) resulted in 3.35%, when mapped against the Baltic lineage (*A. malmogiense*) 4.42%, and against the freshwater lineage (*A. aciculiferum*) 2.86%.

**Supplementary Table 1**. List of *Apocalathium* population origin and habitat.

| **Population label** | **Cell type isolated** | **Origin** | **Country** | **Habitat** | **Salinity** | **Coordinates** | **Nr. of**  **strains** |
| --- | --- | --- | --- | --- | --- | --- | --- |
| SCA-Copenhagen | Motile cell | Sankt Jorgens Sjø | Denmark | Freshwater | - | 55.689362 N 12.579007 E | 14 |
| SCA-Erken | Motile cell | Lake Erken | Sweden | Freshwater | - | 59.85 N  18.63 E | 23 |
| SIB-Baikal | Motile cell | Lake Baikal | Russia | Freshwater | - | 52.2325 N  106.3225 E | 12 |
| BAL-Tvärminne | cyst | Baltic Sea | Finland | Brackish-marine | 6.5 | 59.850985 N  23.260040 E | 22 |
| BAL-Gulf of Finland | cyst | Baltic Sea | Finland | Brackish-marine | 6.5 | 59.5121 N  24.5026 E | 20 |
| ANT-Highway | Motile cell | Highway Lake | Antarctica | Saline lakes | 7.8 | 68.4613889 S  78.22333335 E | 19 |
| ANT-McNeil | Motile cell | Mc Neil | Antarctica | Saline lakes | 7.8 | -68.5277778 S  78.36222227 E | 20 |
| ANT-Vereteno | Motile cell | Vereteno | Antarctica | Saline lakes | 7.8 | -68.515 S  78.4141667 E | 20 |

**Suppplementary Table 2.** Gene ontology annotation of sequences with matches between RADSeq loci and RNA transcripts that are unique for freshwater or saline populations. For stringency only GO terms with at least 10 loci were considered.

| GO term | GO Annotation | Freshwater counts | Saltwater counts |
| --- | --- | --- | --- |
| F:0005247 | voltage-gated chloride channel activity | 0 | 34 |
| F:0005254 | Chloride channel activity | 0 | 29 |
| C:0034707 | Chloride channel complex | 0 | 25 |
| F:0005381 | iron ion transmembrane transporter activity | 0 | 22 |
| F:0015093 | ferrous iron transmembrane transporter activity | 0 | 22 |
| P:0034755 | iron ion transmembrane transport | 0 | 22 |
| P:0072511 | divalent inorganic cation transport | 0 | 22 |
| C:0033573 | high-affinity iron permease complex | 0 | 20 |
| P:0006268 | DNA unwinding involved in DNA replication | 0 | 20 |
| F:0008886 | glyceraldehyde-3-phosphate dehydrogenase (NADP+) (non-phosphorylating) activity | 0 | 19 |
| P:0031460 | glycine betaine transport | 0 | 17 |
| P:0009115 | xanthine catabolic process | 0 | 17 |
| C:0033177 | proton-transporting two-sector ATPase complex, proton-transporting domain | 0 | 17 |
| F:0004855 | xanthine oxidase activity | 0 | 17 |
| C:0033179 | proton-transporting V-type ATPase, V0 domain | 0 | 17 |
| P:0071705 | nitrogen compound transport | 0 | 17 |
| P:0009863 | salicylic acid mediated signaling pathway | 0 | 17 |
| F:0004854 | xanthine dehydrogenase activity | 0 | 17 |
| F:0043047 | single-stranded telomeric DNA binding | 0 | 16 |
| P:0015847 | putrescine transport | 0 | 16 |
| F:0015489 | putrescine transmembrane transporter activity | 0 | 16 |
| P:0007004 | telomere maintenance via telomerase | 0 | 16 |
| P:0006546 | glycine catabolic process | 0 | 16 |
| C:0005891 | voltage-gated calcium channel complex | 0 | 16 |
| F:0015199 | amino-acid betaine transmembrane transporter activity | 0 | 16 |
| F:0042301 | phosphate ion binding | 0 | 16 |
| P:0009870 | innate immune response-activating signal transduction | 0 | 15 |
| C:0000220 | vacuolar proton-transporting V-type ATPase | 0 | 14 |
| P:0052542 | defense response by callose deposition | 0 | 14 |
| P:0052544 | defense response by callose deposition in cell wall | 0 | 14 |
| P:0009620 | response to fungus | 0 | 14 |
| P:0009965 | leaf morphogenesis | 0 | 14 |
| C:0033270 | paranode region of axon | 0 | 13 |
| F:0015204 | urea transmembrane transporter activity | 0 | 13 |
| P:0015840 | urea transport | 0 | 13 |
| F:0004781 | sulfate adenylyltransferase (ATP) activity | 0 | 13 |
| P:0015848 | spermidine transport | 0 | 13 |
| F:0004375 | glycine dehydrogenase (decarboxylating) activity | 0 | 13 |
| P:0048316 | seed development | 0 | 13 |
| F:0015606 | spermidine transmembrane transporter activity | 0 | 13 |
| P:0071918 | urea transmembrane transport | 0 | 13 |
| F:0102067 | geranylgeranyl diphosphate reductase activity | 0 | 13 |
| P:1903711 | spermidine transmembrane transport | 0 | 13 |
| F:0016628 | oxidoreductase activity, acting on the CH-CH group of donors, NAD or NADP as acceptor | 0 | 13 |
| P:0051188 | Obsolete GO term. Please ignore! | 0 | 13 |
| P:0006296 | nucleotide-excision repair, DNA incision, 5'-to lesion | 0 | 12 |
| P:0007608 | sensory perception of smell | 0 | 12 |
| C:0033115 | cyanelle thylakoid membrane | 0 | 12 |
| P:0034502 | protein localization to chromosome | 0 | 12 |
| P:0006906 | vesicle fusion | 0 | 12 |
| C:0034060 | cyanelle stroma | 0 | 12 |
| P:0033137 | negative regulation of peptidyl-serine phosphorylation | 0 | 12 |
| P:0019288 | isopentenyl diphosphate biosynthetic process, methylerythritol 4-phosphate pathway | 0 | 12 |
| P:0030593 | neutrophil chemotaxis | 0 | 11 |
| P:0043525 | positive regulation of neuron apoptotic process | 0 | 11 |
| P:0032729 | positive regulation of interferon-gamma production | 0 | 11 |
| P:1901844 | regulation of cell communication by electrical coupling involved in cardiac conduction | 0 | 11 |
| P:0019740 | nitrogen utilization | 0 | 11 |
| F:0003885 | D-arabinono-1,4-lactone oxidase activity | 0 | 11 |
| P:0032743 | positive regulation of interleukin-2 production | 0 | 11 |
| C:0034704 | calcium channel complex | 0 | 11 |
| P:0035459 | vesicle cargo loading | 0 | 11 |
| P:0008283 | cell population proliferation (for multicellular organisms) | 0 | 11 |
| P:0072383 | plus-end-directed vesicle transport along microtubule | 0 | 11 |
| P:1901898 | negative regulation of relaxation of cardiac muscle | 0 | 11 |
| F:0016899 | oxidoreductase activity, acting on the CH-OH group of donors, oxygen as acceptor | 0 | 11 |
| P:0060314 | regulation of ryanodine-sensitive calcium-release channel activity | 0 | 11 |
| P:0006939 | smooth muscle contraction | 0 | 11 |
| P:0010584 | pollen exine formation | 0 | 11 |
| P:0061028 | establishment of endothelial barrier | 0 | 11 |
| P:0032754 | positive regulation of interleukin-5 production | 0 | 11 |
| P:2000179 | positive regulation of neural precursor cell proliferation | 0 | 11 |
| P:0010880 | regulation of release of sequestered calcium ion into cytosol by sarcoplasmic reticulum | 0 | 11 |
| F:0004001 | adenosine kinase activity | 0 | 10 |
| P:0001955 | blood vessel maturation | 0 | 10 |
| P:0007492 | endoderm development | 0 | 10 |
| F:0008134 | transcription factor binding | 0 | 10 |
| C:0035974 | meiotic spindle pole body | 0 | 10 |
| P:0006614 | SRP-dependent cotranslational protein targeting to membrane | 0 | 10 |
| P:0010273 | detoxification of copper ion | 0 | 10 |
| P:0008219 | cell death | 0 | 10 |
| P:1900245 | positive regulation of MDA-5 signaling pathway | 0 | 10 |
| F:0046429 | 4-hydroxy-3-methylbut-2-en-1-yl diphosphate synthase activity | 0 | 10 |
| P:0006534 | cysteine metabolic process | 0 | 10 |
| P:0051151 | negative regulation of smooth muscle cell differentiation | 0 | 10 |
| P:0023052 | signaling | 0 | 10 |
| P:0010259 | multicellular organism aging | 0 | 10 |
| P:0046688 | response to copper ion | 0 | 10 |
| P:0007596 | blood coagulation | 0 | 10 |
| C:0031307 | integral component of mitochondrial outer membrane | 0 | 10 |
| P:0003096 | renal sodium ion transport | 12 | 0 |
| P:0055074 | calcium ion homeostasis | 12 | 0 |
| P:0035812 | renal sodium excretion | 12 | 0 |
| P:0003091 | renal water homeostasis | 14 | 0 |
| P:0075522 | IRES-dependent viral translational initiation | 24 | 0 |
| P:0032790 | ribosome disassembly | 24 | 0 |
|  |  |  |  |

**Supplementary Table 3.** Summary of retained SNP-loci from Stacks-population runs with varying p (populations required to call a locus) and r (proportion of strains that need to contain the locus for it to be called). Four different *a priori* population divisions were made to explore shared loci. *salinity* (2 populations): saline (all Antarctic+Baltic Sea strains) and freshwater (Scandinavian lakes, Lake Baikal); *species* (3 populations): *A. malmogiense* (Baltic + Antarctic), *A. aciculiferum* (Scandinavian lakes), *Apocalathium* sp*.* (Lake Baikal); *geography* (4 populations): Antarctic lakes, Scandinavian lakes, Baltic Sea, and Lake Baikal. Antarctica denotes Antarctic lakes only.

| Name of population division | Number of populations | Number of populations required (p) | Proportion of individuals with locus required (r) | Retained loci |
| --- | --- | --- | --- | --- |
| Lakes/sites | 8 | 8 | 0.3 | 214 |
| Lakes/sites | 8 | 8 | 0.5 | 35 |
| Lakes/sites | 8 | 6 | 0.4 | 751 |
| Lakes/sites | 8 | 6 | 0.5 | 345 |
| Salinity | 2 | 2 | 0.5 | 123 |
| Species | 3 | 2 | 0.5 | 1846 |
|  | 3 | 3 | 0.5 | 81 |
| Geography | 4 | 3 | 0.5 | 388 |
|  | 4 | 4 | 0.5 | 61 |
| Antarctica | 3 | 3 | 0.2 | 160953 |
|  |  |  | 0.4 | 87957 |
|  |  |  | 0.5 | 58794 |
|  |  |  | 0.6 | 33905 |
|  |  |  | 0.8 | 4262 |
